# Towards a complex ecology: an essay on plague history in Brazil (1890s-1970s)

**DOI:** 10.30827/dynamis.v45i1.33090

**Published:** 2025

**Authors:** Matheus Alves Duarte da Silva

**Affiliations:** * University of St Andrews United Kingdom

**Keywords:** Disease Ecology, Third Plague Pandemic, Brazilian Plague National Service, Brazilian backlands, epidemiological reasoning

## Abstract

This paper offers a periodization of the history of plague in Brazil. It is based on the ways in which experts and public health officers framed the disease, the elements they considered responsible for its spread, and changes in these elements over time. In accordance with this periodization, the article first argues that the ecology of plague became progressively more complex in the 20th century, suggesting the rise of a more ecological-oriented view among Brazilian doctors. It then proposes that political and institutional transformations also shaped this intellectual change in the epidemiological reasoning about pla gue in Brazil. The periodization is divided into three phases. The first phase ex tends from 1897, with the start of discussions about the risk of plague arriving in Brazil from Asia, to 1920, with a substantial reduction in the number of plague cases in coastal cities. In this initial phase, the framing of the plague transitioned from a disease spread by humans and the objects they touched to one spread by rats and their fleas. The second phase, from 1920 to 1950, was characterized by the hegemony of rats in epidemiological explanations for the presence of plague in cities and rural areas of Brazil. The third and final phase, from 1951 to the early 1970s, was characterized by the progressive inclusion of wild rodents into scientific explanations for the spread and especially persistence of plague in some foci, mainly in the North-East. At the end of this phase, the scientific consensus in Brazil was that wild rodents constituted the main plague reservoir.

## 1 Introduction (*)

In 2008 Brazil’s Ministry of Health published its latest official booklet on plague^1^. Plague is no longer a widespread disease in the country: the last human case was registered in 2005, whereas the last deaths date back to 1986^2^. Nonetheless, the risk of plague reappearing in Brazil is not negligible, as the plague bacillus continues to circulate among wild rodents in a few foci. Thereby, the booklet provides important technical information addressed to doctors and other health workers on how to identify a human plague case. Moreover, it describes the complex ecology of plague in Brazil —in other words, the elements, which by their interactions allow the plague bacillus to survive and spread. In Brazil, plague ecology includes several wild rodents, as well as rats, fleas, cats, and humans^3^.

Alongside this medical information, the booklet presents a brief history of plague in Brazil, divided into four main phases^4^. The periodization starts with a “port phase”, existing between 1899 and 1907; then follows an “urban phase” until the 1930s, and a “rural phase”, predominant in the 1930s. Finally, the booklet mentions the current “sylvatic phase”, but the booklet does not indicate when this last phase started. According to the booklet, this last phase is marked by plague siting “in its habitat, constituting natural foci”. In Brazil, most of these natural foci are in a semi-desertic area in the hinterland of the North-East^5^. Therefore, the booklet showcases the history of plague in Brazil as that of a geographical internalization —plague moved from ports around the coast to the hinterland, where it became entrenched among wild rodents.

In the last decades the history of plague in Brazil has also become an object of enquiry for historians. They have commonly focused on three main aspects, starting with the institutional history of the laboratories of Manguinhos and Butantan, created to produce anti-plague serum in the early 1900s^6^. Historians have also paid attention to social, economic and scientific consequences of the arrival of plague in the main Brazilian ports between 1899-1900^7^. Thirdly, they have examined the history of services overseeing the study and control of plague in the North-East of Brazil between the 1940s and 1970s^8^. More recently, some scholars have started paying attention to the global circulation of knowledge on plague having Brazil as one of its nodal points^9^.

Despite their rich contributions, none of these analyses have provided a general interpretation of the history of plague in Brazil in the twentieth century. Surely, such general interpretations can be problematic and arbitrary as they highlight certain points and ignore others, but they can also be helpful by shedding light on historical transformations otherwise impossible to grasp in time-span limited studies. I do not have the pretension of suggesting the ultimate interpretation of the history of plague in Brazil, but rather a possible one. Mine is interested in how the explanations for the epidemiology of plague evolved through the twentieth century in Brazil and how this evolution involved social, political, and epidemiological elements.

In other words, a history of plague that goes beyond a mere geographical displacement from the coast to the hinterland.

To construct this periodization, I will dialogue with two theoretical approaches, starting with the classic studies of Charles Rosenberg on framing diseases. By using the metaphor of frame, Rosenberg argued that a disease is not only a biological entity but a socially constructed one, and that these frames evolved over time^10^. In this sense, I will show the ways in which plague was “framed” in Brazil through the twentieth century by experts and politicians in charge of fighting and controlling it. Moreover, I will dialogue with the more recent works of Christos Lynteris and his ontological approach to plague. As he has showed in several of his studies, the ontology of plague—in other words, what plague “is”— was transformed throughout the last century, when new elements were included in its ecology^11^. In my case, I will show how the “plague frames” ontologically transformed rats and wild rodents in Brazil.

The article is divided in three parts, each showing a particular phase of how plague epidemiology was explained in Brazil. The first section starts in 1897, when Brazilian doctors only associated plague and its transmission with humans and human-made objects. Nonetheless, this interpretation quickly changed, and without necessarily abandoning previous elements, Brazilian doctors began to blame rats and fleas for the spread of plague. The second section discusses the years 1920 to 1950, which were characterized by a dismissal of the role of humans in spreading plague —at least in its bubonic form—, by a hegemony of the rat in the spread of plague, and by the assumption that local wild rodents could be affected by plague, but that this did not constitute an important element of the plague ecology in Brazil. The final section analyses the years spanning from 1950 to the early 1970s, in which rats and fleas became mere intermediary agents in the plague epidemiology, responsible for connecting humans with wild rodents, understood here as the plague reservoir. In short, from a human disease, plague was transformed into a zoonosis affecting mainly rats and secondarily humans, and later, into a disease perpetuated among wild rodents.

By retracing this evolution, I will develop two main arguments. First, that plague did not become a zoonosis only because it began to circulate among rats and later among wild rodents. Instead, political, administrative, and scientific dynamics also played a role in this epistemological transition. Second, although similar transformations on plague epidemiological reasoning were observed worldwide, I argue that this transformation in Brazil had its own logic and chronology, at times going in step with but at times diverging from global scientific dynamics.

### 2 From humans to rats (1897-1920)

In 1894, plague wrought havoc in the British colony of Hong Kong, which would prove to be the beginning of the third plague pandemic^12^. In 1896 the disease reached Bombay (now Mumbai), in India, soon after spreading to all inhabited continents of the world. By the 1950s, it had claimed more than 12 million victims, most in India^13^. In the first five years of the pandemic (1894-1899), important discoveries reshaped the way plague was understood by medical doctors. In 1894, Alexandre Yersin described the micro-organism responsible for it, christened as the plague bacillus, known today as *Yersinia pestis*. Since the first outbreak in Hong Kong rats became associated with the spread of plague^14^. In 1898, Paul-Louis Simond suggested that the rat flea was responsible for transmitting the bacillus from rats to humans^15^. His hypothesis remained controversial in different areas, mainly in India, for a good part of the first decade of the twentieth century, before becoming the main paradigm of the transmission of plague^16^.

Plague started to threaten Brazil in these initial years of the pandemic, which corresponded to the dawn of the First Brazilian Republic (1889-1930). This Republican experience was characterized by federalism and decentralization, which included public health. Every Brazilian state was autonomous vis-à-vis the Union in matters of hygiene, having its own sanitary service and pursuing its independent sanitary politics. The Federal Government had its own sanitary service, the Diretoria Geral de Saúde Publica (DGSP), based in Rio de Janeiro. The DGSP oversaw protecting ports against the so-called exotic diseases and only intervened in provincial public health services if requested. But a judicial and bureaucratic conundrum existed in the Brazilian capital. The city of Rio de Janeiro had a municipal sanitary service. In 1904, the sanitary legislation was partly modified and the DGSP became totally responsible for public health in the Brazilian capital^17^.

Given its role in preventing the importation of “exotic” diseases, DGSP became in charge of protecting Brazil against plague. Targeting the contaminated ports in Asia, DGSP’s director, Nuno de Andrade, banned the importation of objects suspected of carrying plague “contagium” —namely sheets and leather—, ordered disinfections in several other goods, and imposed a 20-day isolation of all passengers upon arrival in Brazil. However, given the distance between Brazil, India, and other plague foci in Asia, most of these measures remained *lettre morte*. But in August 1899, plague outbreaks were officially declared in the cities of Asunción, in Paraguay, and Porto, in Portugal, two ports not only geographically but socially closer to Brazil, as several Portuguese migrants usually departed from Porto. To avoid an invasion, the DGSP imposed sanitary restrictions against Asuncion and Porto akin to those previously applied against Asian ports, which sparked criticisms in the Brazilian press against the rigour of the measures^18^.

Despite these efforts, plague landed in Brazil in the first days of October 1899, in Santos, state of São Paulo, then Brazil’s second-biggest port. Immediately, São Paulo provincial government isolated those contaminated by plague and their relatives and disinfected their houses, while the Federal Government prohibited ships leaving Santos to stop in any other Brazilian port^19^. In January 1900, Rio de Janeiro registered the first confirmed death by plague^20^. From May 1900 the outbreak gathered pace and 295 people died in the Brazilian capital by December^21^. In this first outbreak in Rio de Janeiro, the DGSP intervened in the sanitary services of the capital. DGSP guards isolated patients, disinfected houses, destroyed objects touched by those infected with plague, and promoted anti-plague vaccination^22^. However, the Federal authorities did not impose any restrictions on ships leaving Rio de Janeiro’s port, because this might cause the ruin of the country’s main city and the collapse of the national trade^23^. In sum, believing plague spread by humans, the objects they touched, and by some goods, the first reaction of Brazilian sanitary authorities was to prohibit most of the contacts between confirmed and suspected cases and the rest of the population, while destroying or restricting the circulation of objects considered infected. This strategy was part of a classic pack of measures used in Brazil and other parts of the world to stop contagious diseases, such as cholera and yellow fever^24^.

This strategy suffered an important change at the end of 1899, when the Government of São Paulo began applying anti-rat measures in Santos and the capital, São Paulo. The provincial sanitary service encouraged the population to catch rats and deliver them to the sanitary authorities, in exchange for which they received a small reward. In addition, sanitary officers spread rat poison in the sewage system^25^. The newspaper *Estado de São Paulo* made the connection between plague and rats very explicit, explaining to the public that it was “the fleas of these animals [rats] that communicate this awful disease to man”^26^. Therefore, it was necessary to declare a “war on rats, tenacious and of annihilation” ^27^. These new measures suggest that sanitary officers and journalists of the São Paulo state were aware of research completed in Asia, mainly in India, on possible links between rats and plague. Nevertheless, this new strategy did not imply a complete revolution. Indeed, in further outbreaks in São Paulo state, for instance, in Taubaté, in 1904, and São José dos Campos, in 1906, humans remained seen as agents of the spread of plague. Therefore, those infected by plague, their relatives, and their contacts continued to be isolated^28^.

In Rio de Janeiro, Andrade had initially ruled out a fight against rats, under the double justification that the plague transmission occurred mainly via contact with contaminated persons or objects, and that rat-catching was an expensive and uncertain measure^29^. In March 1903, Andrade was replaced by Oswaldo Cruz as DGSP’s head, as part of a larger plan to modernize and sanitize Rio de Janeiro led by President Rodrigues Alves and Rio de Janeiro’s mayor Pereira Passos^30^. In September 1903, Cruz adopted an anti-plague strategy focused on destroying rats^31^. The DGSP hired squadrons of rat-catchers, known as *ratoeiros*, to purchase rats killed by Rio de Janeiro’s population^32^. Alongside this measure, DGSP agents sulfurized the sewage system, disinfected houses, and removed dead rats from roofs or hidden spaces on the floor. Finally, DGSP pressed landlords to rat-proof their properties, for instance, by filling the gaps between ceilings and wooden floors with cement, where rats supposedly liked to hide and nest^33^.

This change in the anti-plague strategy in Rio de Janeiro was possible, above all, by an increase in the DGSP’s budget, which allowed funding costly anti-rat campaigns^34^. Also, successful anti-rat campaigns inside and outside Brazil provided models to Cruz^35^. Furthermore, there was a general vector turn on the DGSP sanitary strategy to fight epidemic diseases in Rio de Janeiro post-1903. For example, to bring yellow fever under control, Cruz created anti-mosquitos brigades, which focused on the destruction of these insects, their eggs, and larvae^36^. This reorientation was in dialogue with the new international paradigms of microbiology and tropical medicine, according to which many infectious diseases were transmitted to humans by insects like fleas and mosquitos.

Cruz’s anti-rat strategy paid off. From 1903 to 1909, 2,479,782 rats were destroyed and 25,090 houses were turned rat-proof in Rio de Janeiro. In parallel, cases and deaths caused by plague sensibly dropped in the city, passing from 792 and 360, respectively, in 1903, to 40 and 15, respectively, in 1909^37^. In 1912, changes in the command of DGSP, coupled with the costs involved in deratization and the reduction of the presence of plague in Rio de Janeiro, justified replacing this widespread anti-rat campaign with a more focused approach. In this new orientation, DGSP guards caught rats only where human cases or dead rats were found. DGSP continued to isolate human cases and disinfect and rat-proof houses^38^. The revamped strategy remained in place until the end of the 1910s, when plague almost disappeared from the mortuary statistics of Rio de Janeiro^39^.

In short, this first phase of the history of plague in Brazil —corresponding to the fight against the disease in the main Brazilian ports between 1897 and 1920— was characterized by the increasing framing of rats and fleas as the main agents behind the spread of the plague bacillus, which ontologically transformed these animals into public health enemies. Nonetheless, this framing did not discard the possible role that humans could likewise play on it. As summarized by Cruz in 1906, plague was a disease affecting both humans and rats, and transmitted to humans not only by fleas, but also by other humans and the objects they touched^40^. Given this interpretation, the anti-plague strategy adopted mainly in Rio de Janeiro —then plague’s main hotspot in Brazil— became progressively centred on direct and indirect rat destruction, although measures targeting humans remained in place. Conversely, the positive results obtained in fighting rats reinforced the assumption that these animals and their fleas were the main culprits in plague epidemiology.

### 3 Rat hegemony (1920-1950)

The plague pandemic entered a new phase worldwide in the 1920s. The disease shrunk and even disappeared from cities, but advanced towards rural areas. In reason of this displacement, research carried out initially in the USA, South Africa, Manchuria, and the Soviet Union, pointed out that a few local species of wild rodents were also affected by plague and contributed to the survival of the bacillus in these rural areas. This discovery resulted in several new concepts, such as “squirrel plague”, “veld plague”, and “wild rodent plague”, which challenged the centrality of the rat in plague epidemiology^41^. Between 1926 and 1928, the Portuguese doctor Ricardo Jorge coined the concept of sylvatic plague to make sense of all these scattered phenomena of wild rodents being contaminated with and “perpetuating” plague in rural and wild spaces^42^. The Brazilian context of plague studies post-1920 bore connections with this international path, but also significant divergences.

In January 1920, after the disaster caused by the so-called Spanish flu and by the pressing need of sanitizing Brazilian hinterland, the Federal Government extinguished the DGSP and created the Departamento Nacional de Saúde Pública (DNSP). The new department intended to centralize the fight against epidemic and endemic diseases in Brazil, while respecting the autonomy of the states, namely that of São Paulo, in matters of public health. DNSP worked in a system of cooperation: the states that needed federal assistance—virtually all but São Paulo— could ask for it, accepting federal interference. In Getúlio Vargas Era (1930-1945), this centralization of public health increased, which went in pair with a broader process of centralization of the Brazilian state. In 1930, the new regime created a Ministry of Education and Health (in 1953 it was divided into two ministries), which absorbed the DNSP, whose new task became to coordinate public health nationwide^43^.

In the 1920s and 1930s, plague receded from Rio de Janeiro and other coastal cities, advancing towards the hinterland^44^. The deadliest plague outbreak in the 1920s, but potentially of all Brazilian history, occurred in the city of Triunfo, state of Pernambuco. Between 1925 and 1927, more than 2500 people got plague, and around one thousand died, according to some estimations^45^. The Provincial government of Pernambuco, without federal assistance, created the *Inspetoria de Erradicação da Peste*. Directed by Oscar de Britto, the *Inspetoria*’s main goals were the control of plague in Triunfo and other cities in the hinterland of Pernambuco. To achieve this aim, the *Inspetoria* distributed rat traps and fumigated dry walls separating houses and fields, as those were believed to act as rat nests^46^. Britto’s service also studied whether two local wild rodents —*punarés* and *mocós* —were connected to the plague among rats and humans but found no proof in that sense^47^.

After 1930, plague disappeared from most of Brazil but the North-East, where it popped up in small and medium cities, and even in provincial capitals^48^. In 1935, the DNSP organized a series of missions to investigate the presence of plague in that region. Marcelo Silva Junior, one of DNSP plague experts, was sent to Ceará. In his official report published in 1936, he suggested that plague outbreaks in that state seemed somehow connected with wild rodents. This assumption was due both to personal observations and information shared by local informants, according to which epizootics among wild rodents living in uninhabited spaces often preceded epizootics among rats in the cities of Ceará^49^. Nonetheless, Silva Junior did not find any bacteriological proof that those wild rodents epizootics were caused by plague.

One year later, Silva Junior was more successful. In November 1936, he found 14 dead preás, a common rodent, in a scrub (*matagal*, in the original) in the outskirts of the city of Crato, Ceará. He extracted material from these animals and injected with it two guinea pigs. He killed these animals 48 hours later and found that their peritonies were “rich in germs” ^50^. According to Silva Junior, these germs presented physical characteristics that made him think they were the plague bacillus. Also, Crato had been affected by an important plague outbreak in humans in 1936, which had been preceded by a rat epizootic. Drawing upon this evidence, Silva Junior concluded that the *preás* were infected with the plague bacillus and that in Crato plague “followed the cycle *preá*-rat-man [*sic*]”^51^. This conclusion could have important political impacts, as it suggested that the plague bacillus already circulated among wild rodents, at least in this corner of the country^52^.

During these studies, Silva Junior forwarded rodents he collected to the Museu Nacional and fleas to the Instituto Oswaldo Cruz (IOC), both in Rio de Janeiro. In the flea collection, IOC entomologist Angelo Moreira da Costa Lima identified a new subspecies, baptized as *Rhopalopsyllus jordani*, whereas Museu Nacional zoologist João Moojen identified in Silva Junior’s collection a new species of rodent, the *Zygodontis pixuna*^53^. These exchanges marked the debut of a prolific collaboration between doctors, entomologists, and zoologists having the plague among wild rodents in Brazil as its focal point, which would ultimately be central for expanding knowledge on Brazilian fauna and of plague ecology, as discussed in the next section.

Amidst this context of recurrent plague outbreaks in the North-East and doubts about the existence of the plague among wild rodents in Brazil^54^, the Pan-American Health Organization (PAHO) commissioned the Chilean expert Atilio Macchiavello to visit the country. From July 1939 until September 1940, Macchiavello studied the plague situation in the states of Ceará, Paraiba, Pernambuco, Alagoas, and Bahia^55^. In the official report published in 1941, Macchiavello concluded that plague was predominantly rural in Brazil, affecting people living in farms and ranches located at times distant from each other and in areas of scattered population. Repeating an idea already established, Macchiavello affirmed that rats and their ectoparasites were the main villains for spreading and conserving the plague in this rural zone, and therefore, sanitary actions should target them^56^. Macchiavello also observed epizootics among wild rodents such as “mocós”, and “preás”, and among rabbits and hares. He carried out bacteriological examinations on some of the specimens collected already dead in the fields, founding in some the plague bacillus. Macchiavello thus conceded that these animals could be infected and die from plague, but he found no evidence “of a primary wild rodents epizootic”, but quite the opposite: wild rodents were almost infected by domestic rats, because the epizootics among the former proceeded rather than preceded epizootics among the latter^57^. Even though Macchiavello did not discard the likewise of sylvatic plague emerging soon, he considered this was not yet the case^58^. Macchiavello’s scheme was therefore the opposite of Silva Junior’s in 1937: rats and fleas were the reservoir of plague in Brazil, and wild rodents died in most of the cases because they interacted with these animals.

Brazilian sanitary authorities adopted Macchiavello’s reasoning and used it to push for a bigger reform in the fight against plague, resulting in the creation of the *Serviço Nacional de Peste* (Plague National Service, thereafter SNP) in 1941^59^. In his first report as the appointed SNP’s director, Almir de Castro explained the main rationale behind the service’s creation and its future actions:

The path of plague in our country can be divided in three moments: in the first, that of the initial invasion, the disease attacked our main ports; in the second moment, the plague spread through the commercial trade, to the cities of the interior of the country, in the third moment, which is the current one, the disease tends to disappear from the urban environment to be located in rural zones of certain regions, where it can be found today in an endemic form. Fortunately, we have not yet reached the fourth moment, which would be that of the appearance of the sylvatic plague, whose eradication would be impossible. Until now it has not been demonstrated in a conclusive way the existence of sylvatic plague in Brazil^60^.

Castro’s explanation captures the tensions of this second period on the history of plague in Brazil, which had three main characteristics. First, there was a decrease in the importance attributed to humans and objects to the spread of plague. Humans only remained central in the epidemiology of the pneumonic plague, a deadlier type of plague but rare in Brazil^61^. Second, wild rodents, such as the “mocó”, “preá”, and “punarés”, began to be included in the plague ecology, but occupying a peripheral place. Their infection was always provoked by a first contact with rats. Therefore, as its third characteristic, this period was marked by an utterly presence of rats and fleas on plague epidemiological explanations, which was translated into an increased focus on rat destruction.

Different reasons could explain the choice for concentrating the focus on rats and denying the existence of plague among wild rodents in Brazil, or ruling out its epidemiological importance, which made Brazil diverge from other countries where the sylvatic plague was acknowledged, such as USA, Angola, and South Africa^62^. In Brazil, plague experts have not been able to demonstrate that a plague infection among wild rodents was independent from a previous infection among rats. Aside this technical aspect, ruling out the existence of sylvatic plague in Brazil also obeyed a more pragmatic reasoning, partly informed by the discovery of the jungle or sylvatic yellow fever in Colombia and Brazil in the 1930s. In this form, yellow fever circulated among wild animals, namely primates and marsupial living in forested zones, and was transmitted to humans by several mosquitos^63^. The discovery of the sylvatic yellow fever shattered hopes of eradicating yellow fever from Brazil and the Americas by only focusing on eradicating the mosquito *Aedes aegypti*^64^. If the sylvatic plague also existed in Brazil, this would mean that plague eradication was almost impossible, as the bacillus would be circulating among several species of wild rodents^65^. And because eradicating plague was SNP’s main goal, one can then easily understand the effort made by SNP’s members to deny the existence of a local sylvatic plague.

### 4 Wild rodents at the center of plague ecology (1950-1970s)

SNP’s years (1941-1956) overlapped with the last years of Vargas dictatorship (1937-1945) and the first decades of the Third Republic (1945-1964), marked by a huge effort to develop Brazilian hinterland and rural areas. Although the SNP nationalized the fight against plague in Brazil, its focus remained the rural areas of the North-East. In that region, the SNP centred its actions on destroying rats by traps, poisons, fumigation and even flamethrowers. Starting in 1946, SNP began to push homeowners to rat-proof their houses and other buildings, which intended to avoid rats finding shelter and food inside human habitations^66^. Despite the continued centrality of the rat on anti-plague campaigns, SNP did not spare the wild rodents. The SNP forced North-East rural populations to destroy or push away the common life fences (*cercas de aveló*), which separated fields and houses, and were considered a potential point of contact between wild rodents and rats^67^. In 1943 and 1945, SNP’s direction organized specialization courses to its current or potential doctors, with classes about the Brazilian rodent fauna delivered by João Moojen, the rodent expert from the Museu Nacional^68^. This collaboration with the SNP also helped the Museu Nacional to expand its collections — from 1951 to 1955, SNP guards collected around 60,000 specimens of wild rodents, and more than half of these are still conserved in the Museu Nacional^69^.

This interest and continuous studies of wild rodents resulted in the first cracklings in the rat hegemony within the SNP. In 1951, the doctor Roland Simon working on SNP’s laboratory in Maceió, Alagoas state, published an official account on the sensibility of Brazilian wild rodents to plague^70^. In this, he divided the evolution of plague in Brazil on five phases: they were port phase, urban phase, rural phase, “rural-campestre” phase, and sylvatic phase. Simon argued that the rural-campestre phase seemed emergent in the hinterland of the North-East region in the late 1940s. This phase differed from the rural plague described by Macchiavello in 1941, because in the “rural-campestre” phase rats and rodents mutually infected each other at the edges of cultivated and non-cultivated areas, and humans could get plague directly from wild rodents. Nonetheless, this “rural-campestre” phase was not equal to sylvatic plague in Simon’s reasoning, as rats were still the main plague reservoir, and because he imagined that the sylvatic phase could only appear in the jungle. Given this, Simon prophesized that the Amazon, and not the North-East, was the Brazilian region where sylvatic plague was more likely to emerge^71^.

Despite this first attack to the rat hegemony, it was only after the end of the SNP in 1956 that Brazilian doctors began to seriously revise the main intellectual paradigms that underpinned the former service, namely the idea that an independent plague infection among wild rodents did not exist in Brazil^72^. The first study to clearly contest the rat hegemony was published in 1957 by Alberto Gonçalves Neves, formerly attached to the SNP and now to the Departamento Nacional de Endemias Rurais (DNERu), which absorbed the SNP. Neves’ work was based on observations made in a few *sitios* in the state of Ceará, in 1954, and in the state of Pernambuco until February 1956, hence, when the SNP still existed. Neves argued that contrarily to Macchiavello’s interpretation, wild rodents were likely to be the origin of infections among domestic rats. Neves’ reasoning was based on what he called “ecological proofs” and “on epidemiological investigation”. In those *sitios* Neves or some informants, such as farmers and hunters, observed larger epizootics among wild rodents than on domestic rats and sometimes epizootics among wild rodents without epizootics among rats. They also observed some human infections following the manipulation of wild rodents’ carcasses. Neves then concluded that “wild rodents were infected by plague” and that “the nomadism of the domestic rodent links the wild rodents and the man. Everything indicates that the domestic rodent is not the reservoir of plague bacillus”^73^. Nonetheless, cautioned Neves, the “conclusive proof, the bacteriological proof “ was still lacking given the difficulties to ascertain if wild rodents were affected by plague in nature in Brazil^74^.

Contrary to Silva Junior’s 1937 study on sylvatic plague, Neves’ conclusion gained broader support. In 1957 and 1958, PAHO sponsored a mission of another foreign plague expert to Brazil —the Argentinian José Maria de la Barrera, who had a long career on rural and sylvatic plague in South America. In Brazil, De la Barrera concentrated his studies in 13 localities distributed around the states of Bahia, Pernambuco and Ceará. In these places, he focused on the capture of rodents and parasites, sent respectively to the Bernardino Rivadavia Museum in Buenos Aires and Natural History Museum, in England^75^. Among the collected rodents, laboratory investigation identified the presence of the plague bacillus in three specimens found in Brejinho, city of Triunfo^76^. As De la Barrera admitted, this did not proof that the infection among these rodents was not related to a primary infection among rats. However, he pointed out that other wild rodents were found dead in Brejinho, and even if they were not examined, their deaths indicated that a plague infection was going rampant. Moreover, he noted that in Brejinho no epizootic among rats had been detected and that rats captured inside the houses showed no signs of plague. These two facts excluded rats as the cause of plague among wild rodents^77^. Taking these bacteriological, epidemiological, and ecological observations in consideration, De la Barrera affirmed that “the domestic rat (Rattus) does not play a role in maintaining plague, and in Brazil, like in what occurs in other American countries, plague is today limited to the sylvatic fauna of Rodentia and Lagomorpha”^78^. The role played by rats was that of an “intermediary between the sylvatic medium and the man”^79^. Therefore, he concluded that plague in the North-East of Brazil had “the double character of being murine and sylvatic. There are sufficient proofs that the first is a consequence of the second”^80^.

De la Barrera submitted his report in April 1960 to PAHO, which forwarded it to the Brazilian Government in October that year^81^. The report was never published, though, and insofar as sources suggest, it exists only as a dactylography version, which means it only knew a limited circulation among Brazilian and foreign experts. In a synthesis on plague in the Americas sponsored by PAHO, Karl Meyer and Robert Pulitzer agreed with De la Barrera that wild rodents constituted the plague reservoir in Brazil, although they admitted that rats also took part in the epidemiology of plague^82^. In Brazil, on the other hand, De la Barrera’s conclusions on the existence of a wild rodent reservoir of plague reached almost no-impact^83^.

The doubt whether sylvatic plague had emerged in Brazil continued, which justified the creation of the Plano Piloto de Peste (PPP) in 1966, in the city of Exu, state of Pernambuco^84^. The PPP existed until 1974. It was formed by the biologist Célio Rodrigues de Almeida, his then wife, the nutritionist Alzira Maria Paiva de Almeida, and Brazilian and foreign medical doctors. The team was led by Pasteur Institute’s plague expert Marcel Baltazard until his death in 1971. In a series of published works and unpublished reports in the 1970s, Baltazard and the PPP team demonstrated that the plague bacillus circulated among wild rodents —namely among the *pixuna—* independently from previous contacts with rats^85^. In other words, the PPP concluded that sylvatic plague was a reality in Brazil. This time, the existence of the sylvatic plague reservoir became progressively accepted among Brazilian doctors and politicians^86^, and remains the official position of the Brazilian Ministry of Health, as we have seen in the introduction.

This third phase of the history of plague in Brazil, which spanned from 1951 to the early 1970s, was marked by a progressive transition of the roles ascribed to rats and wild rodents in the plague epidemiology. From a peripheral position, wild rodents became framed as the main animals responsible for perpetuating plague in Brazil, whereas rats and fleas remained framed as responsible for spreading it to humans, although direct contacts between humans and wild rodents could also be behind some cases. Therefore, in this last phase the ecology of plague in Brazil reached its current complex form.

## 5 Conclusion

This article is the first to propose a periodization for the history of plague in Brazil based on the evolution of plague epidemiological reasoning in the country. From a disease framed as exclusively linked to humans and objects, plague became framed as a disease affecting mostly wild rodents, at times rats, and accidentally humans. A similar epistemological transition was observed in other parts of the world, from the USA to South Africa. My goal here was not to insist on a Brazilian uniqueness, but to highlight some local aspects that made sense of this epistemological evolution in Brazil. Through the article, I highlighted three main aspects. First, plague disappearing from Brazilian coastal cities and progressively affecting localities in the hinterland pitted doctors against new realities and called for epidemiological explanations that differed from those applied to cities. Second, I have insisted on a progressive nationalization of the fight against plague in Brazil. This allowed plague experts to study the disease beyond the restricted limits of one city or state and pushed them to frame plague as a general phenomenon in Brazil, having different historical phases and a complex ecology. Third, increasing collaborations between medical doctors, biologists, zoologists, and entomologists produced a new understanding, according to which plague epidemiology was a complex web of ecological relations. In short, the evolution of plague epidemiological reasoning in Brazil not only shows how plague ecology became more complex in the scientific explanations of Brazilian doctors, but it suggests the emergence of more ecology-oriented ways of explaining infectious diseases in Brazil, a point to be developed in further publications. ▮

## Footnotes

* Research leading to this article was funded by the Wellcome Trust [grant ID 217988/Z/19/Z] for the project “The Global War Against the Rat and the Epistemic Emergence of Zoonosis”. I would like to thank Mikel Astrain and the *Dynamis* personnel for all your help and support with our special issue!

1 Brasil. Ministério da Saúde, *Manual de vigilância e controle da peste* (Brasília, DF: Ministério da Saúde, Secretaria de Vigilância em Saúde, Departamento de Vigilância Epidemiológica, 2008).

2 Brasil. Ministério da Saúde, 30.

3 Brasil. Ministério da Saúde, 25.

4 Brasil. Ministério da Saúde, 28.

5 Brasil. Ministério da Saúde, 27.

6 Nancy Stepan, *Beginnings of Brazilian Science: Oswaldo Cruz, Medical Research and Policy, 1890-1920* (New York: Science History Publications, 1976); Jaime Benchimol, *Manguinhos do Sonho à Vida: A Ciência na Belle Époque* (Rio de Janeiro: Casa de Oswaldo Cruz, 1990); Jaime Benchimol and Luiz Antonio Teixeira, *Cobras, Lagartos e Outros Bichos: Uma História Comparada dos Institutos Oswaldo Cruz e Butantan* (Rio de Janeiro: Editora da UFRJ/Fundação Oswaldo Cruz, 1993), chap. 1.

7 Henrique Cukierman, “Viagem(ns) a Santos”, *História, Ciências, Saúde-Manguinhos* 5, no. 1 (1998): 35-56; Dilene Raimundo do Nascimento, “La Llegada de la Peste al Estado de Sao Paulo en 1899”, *Dynamis: Acta Hispanica Ad Medicinae Scientiarumque Historiam Illustrandam* 31, no. 1 (2011): 65-83, https://doi.org/10.4321/S0211-95362011000100004; Dilene Raimundo do Nascimento and Matheus Alves Duarte da Silva, “‘Não é Meu Intuito Estabelecer Polêmica’: A Chegada da Peste ao Brasil, Análise de uma Controvérsia, 1899”, *História, Ciências, Saúde-Manguinhos* 20, no. suppl 1 (30 November 2013): 1271-85.

8 Celso Tavares, “Análise do Contexto, Estrutura e Processos que Caracterizaram o Plano Piloto de Peste em Exu e sua Contribuição ao Controle da Peste no Brasil” (PhD Thesis, Recife, Fundação Oswaldo Cruz/Centro de Pesquisas Aggeu Magalhães, 2007); Simone Luna, “O Serviço Nacional de Peste e o Controle da Peste Bubônica no Nordeste Brasileiro (1941-1956)” (Master’s Thesis, Rio de Janeiro, Fundação Oswaldo Cruz/Casa de Oswaldo Cruz, 2021).

9 Matheus Alves Duarte da Silva, “Quand la Peste Connectait le Monde: Production et Circulation de Savoirs Microbiologiques Entre Brésil, Inde et France (1894-1922)” (Thèse de Doctorat, Paris, École des Hautes Études en Sciences Sociales, 2020); Shiori Nosaka and Matheus Alves Duarte da Silva, “Plague and the Global Emergence of Microbiology, 1894-1920”, in *Beyond Science and Empire: Circulation of Knowledge in an Age of Global Empires, 1750-1945*, ed. Matheus Alves Duarte da Silva, Thomas A. S. Haddad, and Kapil Raj, Empires and the Making of the Modern World, 1650-2000 (Abingdon, Oxon; New York, NY: Routledge, 2023), 176-95.

10 Charles E. Rosenberg, “Framing Disease: Illness, Society, and History”, in *Explaining Epidemics and Other Studies in the History of Medicine* (Cambridge: Cambridge University Press, 1992), 305-18.

11 Christos Lynteris, “Zoonotic Diagrams: Mastering and Unsettling Human-Animal Relations”, *Journal of the Royal Anthropological Institute* 23, no. 3 (1 September 2017): 463-85, https://doi.org/10.1111/1467-9655.12649; Christos Lynteris, “Tarbagan’s Winter Lair: Framing Drivers of Plague Persistence in Inner Asia”, in *Framing Animals as Epidemic Villains*, ed. Christos Lynteris (Palgrave Macmillan, 2019), 65-90.

12 Anonymous. “La Peste à Hong Kong”, July 1894. Correspondance consulaire et commerciale (1793-1901), sous-série Hong Kong, tome 5, feuilles 105 et 106. Archives du Ministère des Affaires Etrangères (Pôle La Courneuve).

13 Myron J. Echenberg, *Plague Ports: The Global Urban Impact of Bubonic Plague*,*1894-1901* (New York: New York University Press, 2007); Lukas Engelmann and Christos Lynteris, *Sulphuric Utopias: A History of Maritime Fumigation*, Inside Technology (Cambridge, Massachusetts: MIT Press, 2019); Christos Lynteris, *Visual Plague: The Emergence of Epidemic Photography* (Cambridge, Massachusetts: The MIT Press, 2022).

14 Ernest Hankin, “La Propagation de la Peste”, *Annales de l’Institut Pasteur* XII, no. 11 (1898): 705-62.

15 Paul-Louis Simond, “La Propagation de la Peste”, *Annales de l’Institut Pasteur* XII, no. 10 (1898): 625-87; For a critical examination, see Christos Lynteris, “In Search of Lost Fleas: Reconsidering Paul-Louis Simond’s Contribution to the Study of the Propagation of Plague”, *Medical History* 66, no. 3 (July 2022): 242-63.

16 Indian Plague Commission (1898-1899), *Report of the Indian Plague Commission with Appendices and Summary*, vol. V (London: Eyre and Spottiswoode, 1901); Advisory Committee for Plague Investigation in India, “I. Experiments upon the Transmission of Plague by Fleas: Part II. Transference from Rat to Rat”, *The Journal of Hygiene* 6, no. 4 (September 1906): 435-49.

17 Gilberto Hochman, *The Sanitation of Brazil: Nation, State, and Public Health, 1889-1930* (Urbana: University of Illinois Press, 2016).

18 Anonymous, *Jornal do Commercio*, August 1899; Brasil, *Ministério da Justiça e Negócios Interiores. Ministro Epitácio Pessoa. Relatório dos Anos 1900 e 1901 Apresentado ao Presidente da República dos Estados Unidos do Brazil* (Rio de Janeiro: Imprensa Nacional, 1901), 349; Nascimento and Silva, “Não é Meu Intuito Estabelecer Polêmica”.

19 Brasil, *Ministério da Justiça e Negócios Interiores. Ministro Epitácio Pessoa. Relatório dos Anos 1899 e 1900 Apresentado ao Presidente da República dos Estados Unidos do Brazil* (Rio de Janeiro: Imprensa Nacional, 1900), 355-57.

20 Camillo Terni, Emílio Gomes, and Zacarias Franco, “Confirmação Bacteriológica da Peste Bubônica”, *Brazil-Médico* XIV, no. 5 (1st February 1900): 42-43.

21 Henrique Figueiredo de Vasconcellos, “Prophylaxie de la Peste à Rio de Janeiro”, *Annales de l’Institut Pasteur* XXII, no. 10 (1908): 820.

22 “Camillo Terni, “The Plague in Rio de Janeiro”, Report Attached to the Letter from Lord George Francis Hamilton, His Majesty’s Secretary of State for India, to the Government of India” (Rio de Janeiro, 19 September 1900), India Office Records and Private Papers, Cote: IOR/L/E/7/440, British Library.

23 Brasil, *Ministério da Justiça e Negócios Interiores. Ministro Epitácio Pessoa. Relatório dos Anos 1899 e 1900 Apresentado ao Presidente da República dos Estados Unidos do Brazil*, 361-63; Brasil, *Ministério da Justiça e Negócios Interiores. Ministro Epitácio Pessoa. Relatório dos Anos 1900 e 1901 Apresentado ao Presidente Da República dos Estados Unidos Do Brazil*, 313.

24 Sylvia Chiffoleau, *Genèse de la santé publique internationale: de la peste d’Orient à l’OMS* (Beyrouth: Ifpo - Institut français du Proche-Orient, 2012); Jaime Benchimol, *Dos Micróbios aos Mosquitos: Febre Amarela e a Revolução Pasteuriana no Brasil* (Rio de Janeiro: Editora FIOCRUZ/Editora UFRJ, 1999).

25 Anonymous, “Resumo dos Trabalhos do Instituto Bacteriológico de São Paulo (1892-1906). Peste Bubônica”, *Revista Médica de São Paulo* XI, no. 4 (February 1908): 77-78.

26 Anonymous, “Peste Bubônica”, *O Estado de São Paulo*, 5 November 1899, 1.

27 Anonymous.

28 Cursino de Moura, “Peste Bubônica em Taubaté”, *Revista Médica de São Paulo* VII, no. 24 (1904): 578-79; Carlos Meyer, “A Peste em S. José dos Campos”, *Revista Médica de São Paulo* X, no. 6 (1907): 99-104.

29 Nuno de Andrade, “A Peste e o Brazil-Médico”, *Jornal do Commercio*, 4 October 1901.

30 Jaime Benchimol, *Pereira Passos: Um Haussmann Tropical: A Renovação Urbana da Cidade do Rio de Janeiro no Início do Século XX* (Rio de Janeiro: Prefeitura da Cidade do Rio de Janeiro, Secretaria Municipal de Cultura, Turismo e Esportes, Departamento Geral de Documentação e Informação Cultural, 1990).

31 Oswaldo Cruz, “Relatório do Diretor Geral de Saúde Pública”, in *Ministério da Justiça e* *Negócios Interiores. Ministro J. J. Seabra. Relatório dos Anos 1903 e 1904 Apresentado ao Presidente da República dos Estados Unidos do Brazil*. (Rio de Janeiro: Imprensa Nacional, 1904), 19.

32 This initiative gave birth to mocking carnival songs and several criticisms of the likely possibility of people breeding and “importing” rats. See Edgard de Cerqueira Falcão, *Oswaldo Cruz Monumenta Histórica: Tomo 1 a Incompreensão de uma Época: Oswaldo Cruz e a Caricatura* (São Paulo: s. n., 1971).

33 Oswaldo Cruz, *Peste* (Rio de Janeiro: Besnard Frères, 1906); Vasconcellos, “Prophylaxie de la Peste à Rio de Janeiro’; Henrique Figueiredo de Vasconcellos, “La Prophylaxie de la Peste à Rio de Janeiro”, in *Session Extraordinaire d’avril 1910 du Comité Permanent de l’Office International d’Hygiene Publique: Procès-Verbaux des Séances*, by Office International d’Hygiène Publique (Paris: Librairies-Imprimeries Réunis Martinet Dr, 1910), 11-31.

34 On this budget expansion, see Henrique Cukierman, *Yes, Nós Temos Pasteur: Manguinhos, Oswaldo Cruz e a História da Ciência no Brasil* (Rio de Janeiro: Relume Dumará/FAPESP, 2007).

35 Cruz, *Peste*. Cruz admitted having in mind the strategy adopted by US doctors to control plague in the Philippines, but the city of São Paulo, where anti-rat actions started in late 1899, may have also provided another example.

36 Ilana Löwy, *Virus, moustiques et modernité: la fièvre jaune au Brésil, entre science et politique* (Paris: Éd. des Archives Contemporaines, 2001).

37 Vasconcellos, Henrique Figueiredo de. “Brésil. (Rio-de-Janeiro) Cas et Décès de Peste de 1900 à 1909/Rats Incinérés de 1903 à 1909 et de Maison Rendues Impénétrables aux Rats de 1903 à 1909”. Bulletin Mensuel de l’Office International d’Hygiène Publique II, no. 6 (1910): 933-34.

38 Brasil, *Ministério da Justiça e Negócios Interiores. Ministro Rivadavia da Cunha Corrêa. Relatório dos Anos de 1911 e 1912 Apresentado ao Presidente da República dos Estados Unidos do Brazil* (Rio de Janeiro: Imprensa Nacional, 1912), 21.

39 Brasil, *Ministério da Justiça e Negócios Interiores. Ministro Carlos Maximiliano Pereira dos Santos. Relatório dos Anos de 1915 e 1916 Apresentado ao Presidente da República dos Estados Unidos do Brazil* (Rio de Janeiro: Imprensa Nacional, 1916), 88; Brasil, *Ministério da Justiça e Negócios Interiores. Ministro Urbano Santos da Costa Araújo. Relatório dos Anos de 1918 e 1919 Apresentado ao Presidente da República dos Estados Unidos do Brazil* (Rio de Janeiro: Imprensa Nacional, 1919), 99.

40 Cruz, *Peste*, 7-8.

41 George W. McCoy, “Some Features of the Squirrel Plague Problem”, *California State Journal of Medicine* 9, no. 3 (March 1911): 105-9; Wu Lien-Teh, “Plague in Wild Rodents, Including the Latest Investigations into the Rôle Played by the Tarabagan”, in *Far Eastern Association of Tropical Medicine. Transactions of the Fifth Biennial Congress Held at Singapore 1923*, ed. A. L. Hoops and J. W. Scharff (London, 1924), 305-40; James Hunter Harvey Pirie, “Plague on the Veld”, in *The Plague Problem in South Africa: Historical, Bacteriological, and Entomological Studies*, by James Alexander Mitchell, James Hunter Harvey Pirie, and Alexander Ingram (Johannesburg: South African Institute for Medical Research, 1927), 138-62; S. M. Nikanoroff, “Union des Republiques Socialistes Sovietiques”, in Les Faunes Régionales des Rongeurs et des Puces dans leurs Rapports avec la Peste (Deuxième Partie)’: *Résultats de l’enquête du Comité Permanent de l’Office International d’hygiène Publique 1924-1927*, by Ricardo Jorge (Paris: Masson et cia, 1928), 96-123.

42 Ricardo Jorge, “Les Faunes Régionales des Rongeurs et des Puces dans leurs Rapports avec la Peste (Deuxième Partie)”, *Bulletin Mensuel de l’Office International d’Hygiène Publique* XIX, no. 9 (September 1927): 1257-88; Ricardo Jorge, “La Peste Africaine”, *Bulletin Mensuel de l’Office International d’Hygiène Publique* XXVII, no. 9 (Supplement) (1935): 1-67.Jorge, “Les Faunes Régionales des Rongeurs et des Puces dans leurs Rapports avec la Peste (Deuxième Partie)’; Matheus Alves Duarte da Silva, “Between Deserts and Jungles: The Emergence and Circulation of Sylvatic Plague (1920-1950)”, *Medical Anthropology*, 2023.

43 On this progressive centralization, see Hochman, *The Sanitation of Brazil*.

44 Emygdio Mattos, “Peste”, *Archivos de Hygiene* I, no. 1 (1927): 125-43; Decio Parreiras, “Notas e Estudos Sobre a Peste no Nordeste do Brasil, Problema Nacional”, *Archivos de Higiene* V, no. 1 (1935): 45-49; Mario da Camara Motta, “O Problema da Peste no Estado da Parahyba”, *Archivos de Hygiene* VI, no. 1 (1936): 187-210; Marcelo Silva Junior, “Peste no Ceará”, *Archivos de Hygiene* VI, no. 1 (1936): 155-86.

45 Marcelo Silva Junior, *Peste Bubônica* (Rio de Janeiro: Jornal do Commercio, 1942), 20-21.

46 Oscar de Britto, *Serviço de Erradicação da Peste em Triumpho* (Recife: Estado de Pernambuco. Departamento de Saúde e Assistência Pública, 1929), 18.

47 Britto, 14.

48 Parreiras, “Notas e Estudos Sobre a Peste no Nordeste do Brasil, Problema Nacional”.

49 Silva Junior, “Peste no Ceará”, 177.

50 Marcelo Silva Junior, “Peste Silvestre no Ceará”, *Arquivos de Higiene* VII, no. 1 (1937): 95-96.

51 Silva Junior, 96.

52 It should be noted that Silva Junior’s conclusion could appear slightly odd, as he found dead *preás* after rats and human died of plague, but concluded the other way round, that preás were infected first. How to make sense of this contradiction? Perhaps, Silva Junior was suggesting that the epizootic among the preás was the beginning of new epidemiological cycle in Crato, in which an epizootic among rats would follow.

53 Marcelo Silva Junior, *Conclusōes Finais de 3 Anos de Estudos em Torno do Problema da Peste no Nordeste* (Rio de Janeiro: Canton & Reiler, 1943), 6 and 14-16.

54 In further research, Silva Junior demonstrated that some wild rodents could die of plague experimentally in the laboratory, and that in nature some specimens also got contaminated. However, in two studies published in 1942 and 1943, he abjured his previous conclusion, affirming, instead, that plague epizootics among wild rodents were rare in Brazil, and always dependent of a previous infection among domestic rats. See João Moojen and Marcelo Silva Junior, “Roedores Domesticos e Silvestres: Sua Relaçao com a Peste e Normas Gerais da Respectiva Caracterização Específica”, *Archivos de Hygiene* Anno 12, no. 2 (1942): 165; Silva Junior, *Conclusōes Finais de 3 Anos de Estudos em Torno do Problema da Peste no Nordeste*, 18.

55 Atilio Macchiavello, *Contribuciones al Estudio de la Peste Bubonica en el Nordeste del Brasil* (Guayaquil: Pan American Sanitary Bureau, 1941), 9-10.

56 Macchiavello, *Contribuciones*.

57 Atilio Macchiavello, Hélio Paracampos, and Celso Arcoverde Freitas, “Peste Espontanea en Animales Silvestres”, in *Contribuciones*.

58 Macchiavello, *Contribuciones*, 103.

59 Almir de Castro, “Atividades de Profilaxia Antipestosa do Departamento Nacional de Saúde do Ministério da Educação e Saúde 1937-1941” (Rio de Janeiro, 31 October 1942), 1-2, GC h 1940.03.11, Fundação Getúlio Vargas.

60 Castro, 1-2.

61 Mattos, “Peste”, 129-30; Celso Arcoverde Freitas and Valença Junior, “Peste Pneumônica em Pesqueira”, *Revista de Higiene e Saúde Pública* XIV, no. 4 (1955): 73-78.

62 Pirie, “Plague on the Veld’; Francisco Venáncio da Silva, *Serviço Permanente de Prevenção e Combate à Peste Bubônica no Sul de Angola/ Relatório 1933* (Lisboa: Divisão de Publicaçōes e Biblioteca. Agencia Geral das Colônias, 1936); Karl F. Meyer, “The Sylvatic Plague Committee”, *American Journal of Public Health and the Nation’s Health* 26, no. 10 (October 1936): 961-69.

63 Fred L. Soper *et al*., “Yellow Fever without Aëdes Aegypti. Study of a Rural Epidemic in the Valle Do Chanaan, Espirito Santo, Brazil, 1932”, *American Journal of Epidemiology* 18, no. 3 (1933): 555-87; Fred L. Soper, “The Newer Epidemiology of Yellow Fever”, *American Journal of Public Health and the Nations Health* 27, no. 1 (1937): 1-14; Evandro Chagas, “Exposição Feita ao Superintendente do Serviço de Estudo das Grandes Endemias, pelo Dr Atilio Macchiavello, em Nome do Dr John Long, Representante da Oficina Sanitaria Panamericana no Brasil para o Problema da Peste Humana” (Rio de Janeiro, 1 July 1940), GC h 1940.03.11, Fundação Getúlio Vargas.

64 Emilio Quevedo *et al*., “Knowledge and Power: The Asymmetry of Interests of Colombian and Rockefeller Doctors in the Construction of the Concept of “Jungle Yellow Fever,” 1907-1938”, *Canadian Bulletin of Medical History* 25, no. 1 (April 2008): 71-109; Rodrigo Cesar da Silva Magalhães, *A erradicação do Aedes aegypti: febre amarela, Fred Soper e saúde pública nas Américas (1918-1968)* (SciELO - Editora FIOCRUZ, 2016), 96-100.

65 Castro, “Atividades de Profilaxia Antipestosa do Departamento Nacional de Saúde do Ministério da Educação e Saúde 1937-1941”, 1-2.

66 Luna, “O Serviço Nacional de Peste e o Controle da Peste Bubônica no Nordeste Brasileiro (1941-1956)”, chap. 3.

67 Celso Arcoverde Freitas, Histórias da Peste e de Outras Endemias, Rio de Janeiro, 1988, 75-76.

68 Freitas, 50-52.

69 João Alves de Oliveira and Stella Maris Franco, “A Coleção de Mamíferos do Serviço Nacional de Peste no Museu Nacional, Rio de Janeiro, Brasil”, *Arquivos do Museu Nacional* 63, no. 1 (2005).

70 Roland Simon, *Verificação da Sensibilidade dos Roedores da Região Neotrópica* (Rio de Janeiro: Gráfica Debret, 1951).

71 Simon, 25-26.

72 In March 1956 a large public health reform took place in Brazil. In this, the SNP was extinguished, and its personnel absorbed by the recently created Departamento Nacional de Endemias Rurais (DNERu). This new department became in charge of the fight against plague and other rural endemic diseases. See Anonymous, “Criação do Departamento Nacional de Endemias Rurais”, *Revista Brasileira de Malariologia e Doenças Tropicais* VIII, no. 2 (1956): 409-14.

73 Alberto Gonçalves Neves, *O Problema da Peste dos Roedores Silvestres no Nordeste Brasileiro* (Rio de Janeiro: Ministério da Saúde. Departamento Nacional de Endemias Rurais. Divisão de Cooperação e Divulgação, [1957] 1965), 18.

74 Neves, 1-2.

75 “Letter from José Maria de la Barrera to Franciscus Smit” Recife (Brazil), 20 June 1957), Smit Correspondence, BA-BL, DF 340/2, Barrera, Prof José Maria de la Barrera (II), Natural History Museum (London) Archives.

76 José Maria de la Barrera, “Relatório Sobre a Peste no Brasil” (April 1960), 101, Museu Nacional, Setor de Vertebrados.

77 De la Barrera, 108.

78 De la Barrera, 148.

79 De la Barrera, 146.

80 De la Barrera, 141.

81 De la Barrera, 101. I am very thankful to Professor João Alves de Oliveira for sharing with me his version of the report.

82 PAHO, *Plague in the Americas* (Washington: PAHO, 1965), 51 and 64.

83 Grupo de Trabalho, “Campanha Contra a Peste” (1960), 147, Personal Archives - Alzira de Almeida.

84 On this and other points of the history of the PPP, see Tavares, “Análise”, 51-52.

85 Marcel Baltazard, “Recherches sur la Peste au Brésil. Quatrième Rapport.” (Paris, 1970), Personal Archive - Alzira de Almeida; Célio Rodrigues de Almeida and Alzira Maria Paiva de Almeida, “Le Probleme de la Peste au Brésil” (Paris, 1976), Personal Archives - Alzira de Almeida.

86 See, for example, SUCAM, “Reuniao de Grupo de Peste-1977” (Guaranhuns, 1977), Personal Archive - Alzira de Almeida. Interestingly, the conclusions by the PPP team on the existence of a sylvatic plague in Brazil were fiercely criticised by Marcelo Silva Junior. See Marcelo Silva Junior, “Mesa Redonda Sobre Peste - IX Congresso da Sociedade Brasileira de Medicina Tropical - Fortaleza - Ceará – 04 a 08/02/1973. Participaçao do Dr. Marcelo Silva Junior” (Fortaleza, 1973), Personal Archives - Alzira de Almeida.
